# Natural killer cells in skin: a unique opportunity to better characterize the many facets of an overlooked secondary lymphoid organ

**DOI:** 10.3389/fimmu.2025.1646719

**Published:** 2025-08-12

**Authors:** Kirsten M. Johnson, Dean A. Lee

**Affiliations:** ^1^ Department of Dermatology, College of Medicine, The Ohio State University Wexner Medical Center, Columbus, OH, United States; ^2^ Division of Hematology/Oncology, Nationwide Children’s Hospital, Columbus, OH, United States

**Keywords:** natural killer cells, innate lymphoid cells, cutaneous disease, skin, innate immunity

## Abstract

Natural killer (NK) cells are lymphoid-derived cells that play a critical role in bridging innate and adaptive immunity. Given their ability to recognize and directly kill targets possessing missing or altered self-proteins and to induce indirect killing via recruitment of adaptive immunity, they are in a unique position to modulate host immunologic responses. These complex immune sentinels typically circulate in the peripheral blood and/or reside in lymphoid tissues. As the largest organ, human skin functions in front line immunological defense, though it has not historically been categorized as lymphoid tissue. Whether tissue-resident ILC populations originally derive from conventional circulating NK cells, or whether they interface as developmentally distinct entities with phenotypic overlap within particular inflammatory contexts remains a subject of ongoing investigation. This review seeks to consolidate the currently available literature regarding NK cell and ILC skin homing and innate immune function in healthy vs. lesional human skin (including infection, inflammatory/autoimmune conditions, and cutaneous malignancy). Importantly, we elucidate significant gaps in the understanding of the complex role for NK cells in skin homeostasis and pathology, and posit unique opportunities the accessibility of this secondary lymphoid organ provides for translational studies to improve our understanding of cutaneous immunity.

## Background

NK cells are immune effectors of the ILC family that play critical roles in both innate and adaptive immunity. They are generally found circulating in peripheral blood, but are recruited to specific tissues in response to stress signals initiated following host recognition of pathogenic cells, especially viruses and tumors (1). With their unique ability to recognize missing or altered self-proteins, or overexpressed non-self-proteins, NK cells play a critical role in eliminating pathologic cell populations through both direct and indirect mechanisms. These lethal cells eliminate their targets through both direct and indirect means. NK cells mediate direct killing via cytotoxic granule exocytosis, and indirectly by inducing death-receptor-mediated apoptosis via Fas or tumor necrosis factor (TNF) ligands (2). They also mediate antibody-dependent cellular toxicity, induced by NK cell expression of the Fc receptor CD16 (3). Additionally, NK cells secrete interferon (IFN)-γ and TNFα, which activate other immune targets crucial for initiating the adaptive immune response (4).

Members of the ILC family are typically differentiated by their cytokine secretion signature and transcription factor response (5) (**Table 1**) (6–45). For example, NK cells circulating in the peripheral blood are dependent on the transcription factor eomesodermin (Eomes) and secrete TNFα, IFNγ, perforin, and granzyme B upon activation (6, 7, 46).While these “conventional” NK cell (cNK) populations circulate in the blood, the remainder of the ILC subsets (ILC1, ILC2, ILC3, and lymphoid tissue inducer (LTi) cells) tend to be tissue-resident and demonstrate differential transcription factor dependencies associated with particular tissue localization (17). cNK cells are defined by expression of NK1.1, Nkp46, and CD49b in mice (47), whereas tissue-resident ILC1 cells express CD49a, which promotes lymphocyte homing to non-lymphoid tissues, and CD69, which retains lymphocytes within the tissue (6, 19, 48–50). A recent study demonstrated remarkably conserved expression of CD56^bright^Tcf7^hi^CD69^hi^ tissue resident NK cells in both murine and human skin (14), making murine models a helpful and generally translatable means of studying the role of NK cells in skin pathology. While CD69 is a marker of tissue residency, Tcf7 heralds stemness and is typically down-regulated in tissue-resident T-cells (51). Similarly, CD56^dim^ NK cell populations also appear to be conserved between mice and humans and are typically isolated from circulation (14, 52).

**Table 1:** NK cell vs ILC cell subsets.

Identifiers	cNK	ILC1	ILC2	ILC3	trNK
Transcription factors	Eomes (6, 7) T-bet (8, 9)	Hobit (10) T-bet (9, 11)	GATA3 (9, 12)	RORγT (9)	+/- Eomes & Tbet (13), Tcf1 (14)
Chemotactic and homing molecules	CCL3, CCL4, and CCL5 (15, 16) XCL1 (17, 18), XCL2 (18)	CCL4 (19) CXCR3 and CXCR6 (20, 21), XCL1 (17), CXCL9 (22)		CCR6 (11)	CD69 (13, 14), CCR5 (liver) (23), CXCR6 (liver but not skin) (23),
Cytokines	IFNγ (24–26), TNFα (16, 24, 27), GM-CSF (16)	IFNγ (11, 28), TNFα (11)	IL-4, IL-5, IL-9, & IL-13 (12), IL-10 (29), Amphiregulin (30)	IL-17 (31), IL-22 (31, 32), GM-CSF (33), TNFα (34), LTa & LTb (34, 35), BAFF (36)	IFNγ (murine) (13, 14), TNFα & GM-CSF (13)
Other Effectors	CD16→ADCC (3) Granzyme/perforin (37) TRAIL/FASL (38–40)	Granzyme (10, 19) TRAIL (41, 42)	PGD2 (43)	RANKL (36, 44)	TRAIL (45)

A recent hallmark study made great strides in this classification dilemma via integration of robust scRNAseq and CITEseq data from healthy human blood, tissues (specifically lungs, intestinal epithelium, and tonsils), and a number of tumors (53). Their data identified three distinct NK cell subsets within human blood: NK1, NK2, and NK3 cells, each representing a different state of “maturity” and distinct capacity for the various inherent NK cell functions (cytotoxicity, cytokine secretion/proliferative capacity, and adaptability, respectively). In the majority of the tumors analyzed, the proportion of NK2 cells (CD56^bright^CD16^neg^) were increased at the tumor bed compared to peripheral blood, with a bias in their innate transcriptional profile toward expressing genes implicated in cell migration and tissue homing (53). As discussed by Chaudhry et al., NK cells entering tissues appear to take on distinct phenotypes and adopt responsiveness to tissue-specific transcriptional programs upon entry. This plasticity can make it difficult to distinguish NK from tissue-resident ILC1 cells in the different tissue types, such as in a subset of NK cells in human lung that express the classically associated transcription factor Hobit (54). In contrast, while Hobit is also expressed by ILC1s in mouse lamina propria (10, 55), intestinal ILC1 cells in humans instead express Prdm1 (Blimp1) (56). These findings are yet to be characterized in skin.

NK cells are typically subdivided into two groups based on their expression of CD56 and CD16. Generally, CD56^bright^CD16^neg^ NK cells are thought to have an immunoregulatory phenotype (57–60). These cells are commonly found in secondary lymphoid organs and display poor cytolytic and ADCC capacity, but rapidly secrete cytokines and chemokines; these are also highly responsive to cytokine secretion within the tissue microenvironment [reviewed in 61]. In contrast, CD56^dim^CD16^+^ NK cells comprise 90% of all NK cells in circulation and are characterized by potent cytolytic function and the capacity to robustly mediate ADCC, but with the tradeoff of a more limited cytokine secretion repertoire (61–63). Though controversial, trNK cells are regarded by some as a subset of group 1 ILCs, which includes ILC1 cells and conventional/peripheral NK cells. In other models, resident NK cells exist on a spectrum with ILC1 cells. Some studies suggest cNK cells can differentiate into ILC1-like cells via IL-12 and TGFβ signaling following suppression of Eomes expression, with a simultaneous increase in T-bet expression (46). This transdifferentiation process appears to provide at least one strategy for tumor immunoevasion, mediated by tumor-secreted TGFβ inducing NK signaling via a non-canonical Smad4 independent pathway (64, 65).

Human skin consists of three distinct layers—the outermost epidermis, the dermis, and the underlying subcutis. The epidermis primarily provides a physical barrier function, while the dermis is traversed by blood vessels and lymphatics, allowing recruitment of both innate and adaptive immune cells that facilitate its participation in cell-mediated immunity (66). Additionally, the dermis contains complex networks of collagen and elastin fibers, collectively forming the extracellular matrix (ECM). This critical scaffold affects both recruitment and migration of immune cells into specific tissues (67). Much of the investigation to date regarding innate immunity in the skin focuses on identifying specific ILC subsets, with more recent studies evaluating tissue resident NK (trNK) cells. Key questions to address include whether trNK cells arise during skin development, or do they migrate from circulation in response to pathogen-specific exposures? Also, what is the role of trNK cells in specific cutaneous pathologies?

## Skin trNK cell origin—current understanding

To date, most studies of NK cell populations in the skin have focused on ILC subsets (usually categorized as ILC1, ILC2, and ILC3 cells; see **Table 2** (68–80)). Though found in every tissue, ILCs are especially prevalent in barrier tissues, such as the skin, lung, and intestines, where they are thought to be important for maintenance of tissue homeostasis, as well as mediating initial inflammation in response to pathology such as infection (81). A number of insightful review articles have outlined the role and contribution to skin pathology of these ILC subsets (46, 81–83). Notably, a recent review of trNK cells included a discussion of their presence and function in every organ known to house NK cells *except* the skin (84). Single cell transcriptomic profiling of developing fetal and adult healthy human skin compared with skin from adult patients with atopic dermatitis and psoriasis demonstrated an enrichment of ILCs within the skin during the first trimester for fetal tissue, as well as clonal expansion of disease-associated lymphocytes in diseased skin (85). In mice, NK 1.1+ ILC1 cells are highly enriched in fetal and neonatal skin, but rapidly decline within 6 weeks of life (86). These cells are believed to play a significant role in regulating early cutaneous microbiota colonization. Another study in mice demonstrated a population of invariant natural killer T (iNKT) cells that enter the skin early in the post-natal period, and play a crucial role in skin tissue development and homeostasis through transferrin-mediated regulation of iron metabolism (87). Given nomenclature variability, it is unclear whether these iNKT cells are related to a population of “epidermal lymphoid cells” that express NK cell markers and secrete IFNγ upon stimulation that have been identified seeding the epidermis during late embryogenesis in both wild type and T-cell deficient mice (88).

**Table 2:** Surface markers of NK cell vs ILC subsets in peripheral blood vs skin.

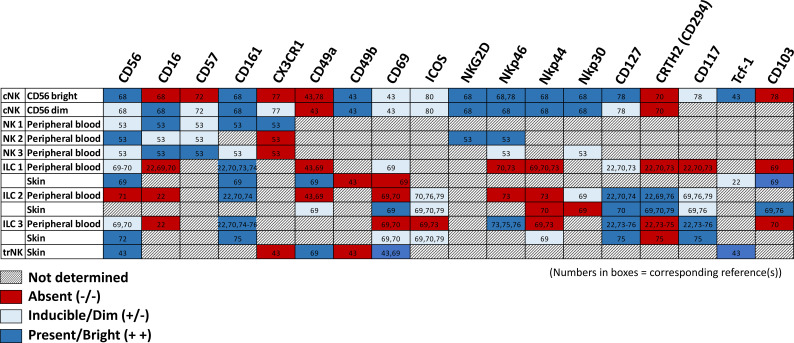

All three ILC subsets are present in healthy human skin (89, 90); however, they are differentially expressed in disease-specific states (e.g., ILC3s are significantly increased in lesional psoriasis) (91). scRNA-seq of lesional vs non-lesional skin identified four unique clusters of innate lymphocytes in skin that were CD161(KLRB1)^+^/CD3(CD3D/CD3G)^neg^. These subgroups included ILC1/3, ILC2, ILC1/NK cells, and NK (KLRD1^+^, GNLY^+^, PRF1^+^, GZMB^+^, and FCGR3A^+^) cells (85). In healthy human and murine skin, most effector ILCs are of the ILC2 subset, which produces IL-5 and IL-13 and are increased in cutaneous inflammatory disease (43, 92). These cells have also been implicated in wound healing (93). Some propose that ILC2 and ILC3 cells are predominantly tissue resident in healthy homeostatic states, whereas ILC1 cells continuously traffic between circulation and lymph nodes in a CD62L- and CCR7-dependent manner (94).

Healthy human dermis contains NK cells that are CD56^+^ and uniformly CD16a^neg^CCR7^neg^, in contrast to circulating NK cells, which mostly express CD16a and frequently CCR7 as well (95). CD16a is an IgG Fc receptor expressed by NK cells that is essential for inducing antibody-dependent cellular toxicity (3). Upon NK cell activation, this receptor is cleaved by metalloproteinase 17 (ADAM-17) (96–98). ADAM-17, a critical upstream regulator of epidermal growth factor receptor (EGFR) signaling that is vital for human development, is ubiquitously expressed, including by keratinocytes (99). Keratinocyte-specific knock out of ADAM-17 results in mice born with a normal epidermal barrier; however, they quickly (within days of birth) develop compromised epidermal integrity and demonstrate chronic dermatitis as adults (100). ADAM17 inhibition in co-culture experiments prevents CD16a shedding by NK cells and increases their cytolytic function against breast cancer cells (101–103). These studies suggest that upon skin entry, trNK cells derived from circulation lose CD16a expression, likely mediated by ADAM-17 cleavage. It is unclear whether this loss may contribute to the identification of NK cells within the dermis, gut lamina, and cryopatches that demonstrate reduced ability to produce IFNγ or to degranulate (1).

Skin parabiont experiments demonstrate that the majority of trNK cells in the skin are very similar to those found in the liver and uterus, possessing CD49a^+^DX5^neg^ markers (13). T-bet knock out mice lack both skin and liver trNK cells, suggesting a common precursor cell that is T-bet dependent, whereas uterine trNK cells are still present in T-bet deficient mice (13). Skin trNK cell development is also independent of HOBIT/ZNF683 (14), a transcription factor known for regulating differentiation of human CD34^+^ progenitor lymphocytic cells into CD56^+^ NK cells (104). Additionally, this differentiation does not appear to require the TGFβ receptor, which plays a central role in mediating development of skin-localized CD8^+^ resident memory T-cells (14).

An older proposed classification for tissue-derived NK cell lineages included two circulating populations and two tissue resident populations: 1) cNK cells in spleen, blood, and other organs, 2) thymic cNK cells, 3) trNK cells in liver and skin, and 4) uterine trNK cells (13). These “liver-resident” NK cells (CD49aEomes-NK1.1+KNp46+), however, are now generally classified as ILC1s, reflecting their distinct developmental origin and functional identity (10, 55). Klose et al., 2014 and Robinette et al. were first to delineate ILC subsets across tissues, highlighting the distinction between ILC1s and NK cells, which derive from different progenitors (11, 42). More recently, Nixon et al. described a population of CD49aEomes+ trNK cells in the salivary gland (“SG-ILC1”) that are phenotypically and developmentally distinct from both liver ILC1s and cNK cells (105). Together, these findings support the existence of at least three distinct Group 1 ILC populations: 1) ILC1, trNK, and cNK cells. Interestingly, trNK cells appear to represent a hybrid lineage with features derived from both ILC1 and cNK cell pathways. Torcellan et al. also recently described a group of skin-resident NK cells that acquire both long-term residency and memory properties in response to but then sustained following resolution of murine skin CMV infection. These were notably distinct from the previously identified ILC1, salivary trNK and cNK cell populations (14). In addition to providing a model by which circulating NK cells may establish tissue residency in the skin, these and prior findings importantly highlight tissue-specific diversity within the Group 1 ILC compartment. Overall, differentiating when and how circulating NK cell populations establish residency versus transiently infiltrate tissue(s) in response to infectious stimuli, remains a topic of ongoing investigation (106).

NK cells identified within skin and other peripheral tissues (unlike those in circulation) lack direct cytotoxic function (107). This absence may be altered/blocked upon exposure to ECM proteins within the dermis, as previously discussed. Further evidence for this alteration was demonstrated through a murine cytomegalovirus (mCMV) model (m157), which acts as an NK cell-specific activating ligand. Bunting et al. demonstrated that mCMV skin grafted onto the back of C57BL/6 WT mice recruited circulating cNK cells, giving rise to trNK cells in donor skin. Both CD49b^+^ cNK and CD49a^+^ trNK cells found in the m157-expressing skin grafts were *recipient* derived (107). Entry into the skin resulted in a drastic change of NK cell function, including down-regulation of their cytotoxic program, while simultaneously boosting chemokine and inflammatory cytokine production (107). Interestingly, trNK cells isolated from donor skin that were then tested *in vitro* regained their cytotoxic ability to fully degranulate *outside* of the skin. In an elegant parabiosis experiment, this group further demonstrated that NKp46^neg^GFP^+^ NK cells injected intravenously into WT mice one day following skin transplantation were found in the recipient liver as well as in the skin graft twenty days later. These findings suggest the NK cells that set up tissue residency in skin grafts are recruited from circulation, then adopt skin tissue resident properties after migration to the dermis/epidermis (107). It also further supports an as yet undefined relationship between skin and liver trNK cells (108).

## NK cell migration to the skin

As mentioned above, innate immune cells are among the most common lymphocytes found within skin in the early post-natal period, consisting predominantly of innate T-cell subtypes (109–111). Importantly, skin colonization with normal bacterial flora during early neonatal development invites an influx of activated regulatory T (Treg) cells into the skin that are critical for establishing immune tolerance to commensal organisms (111). Transcriptomic profiling of immune cells in mouse skin at different post-natal time points demonstrates that iNKT cells are especially dominant and play critical roles in skin development and homeostasis during this early period (87). These innate-like αβ T cells present in infant and young mice are gradually replaced in the early post-natal period by more adaptive immune populations in a microbiota-driven process (87).

T-cell lymphocyte trafficking and entry into the skin is well-characterized (112); however, data regarding NK cells in this arena is limited at best. In general, leukocytes homing to the skin adhere to the endothelium of dermal vasculature via β2 integrins such as LFA-1 (CD11a/CD18, αLβ2) or Mac-1 (CD11b/CD18, αMβ2) binding to ICAM-1 (CD54) (113, 114) or via β1 integrins binding to VCAM-1. Proinflammatory cytokines, such as IFNγ, TNFα, and IL-1, trigger ICAM-1 and VCAM-1 activation to facilitate this T-cell localization to sites of skin inflammation (114–117). The complex structural fortification of the ECM makes further lymphocyte localization to the epidermis difficult (107). Though many chemokines are involved, the CCL17-CCR-4 and CCL27-CCR10 axes appear to be the most influential in recruitment of T cells to active sites of skin inflammation, such as in psoriasis and atopic dermatitis (AD) [reviewed by 112]. Additionally, CCR6 is a key chemokine for directing skin homing of regulatory T cell (Tregs) in psoriasis pathogenesis (112). In contrast, trNK cells in the skin express a variety of chemokine receptors, including high levels of CXCR3 and CCR5, and infiltrate the skin in response to disease-specific chemokines, such as CLL5 in psoriasis (118). Though the phenotypic tissue and memory profiles for recruited T and NK cells generally mirror each other, it is important to characterize their cell-specific recruitment to and maturation within skin tissue, since they maintain distinct programs of differentiation (61).

While CCR10 expression is especially prevalent on skin memory-like resident T cells (119), the majority of ILCs in human skin also express CCR10 (43), which plays a role in recruiting activated lymphocytes from skin-draining lymph nodes to help regulate the homeostasis of T helper (Th) cells and Tregs (120). Irradiated CCR10-/- mice demonstrated lower skin ILC percentages and increased cutaneous damage compared with CCR10+/- controls (121). Specifically, ILC cells found in skin draining lymph nodes of CCR10 knock out mice demonstrate defective migration to the skin and instead divert to other sites lacking expression of CCR10 ligands, such as the lungs (120). CCL27, the ligand for CCR10, is highly and specifically expressed on epidermal basal keratinocytes in both healthy mouse and human skin, and is upregulated during inflammation (122). This ligand is also critical for the establishment of resident lymphocytes in skin and mucosal tissues, independent of stimulation by local commensal microbial populations (123). Another critical receptor in this homing process is cutaneous lymphocyte associated antigen (CLA). CLA is a glycoprotein expressed on the surface of various lymphocyte populations that serves as a homing receptor for skin-infiltrating NK cells (124, 125). It binds to E-selectin, which is expressed in endothelial cells and other tissues during acute inflammation (126). Intra-dermal injection of recombinant human CCL27 immediately recruits CLA^+^ homing lymphocytes from circulation (122). Additionally, dermal microvascular endothelial cells, fibroblasts, and circulating CLA^+^ T cells demonstrate surface expression of CCR10, all of which suggests a critical role for the CCR10-CCL27 axis in recruitment and migration of lymphocyte populations directly to skin (122). While skin infiltration also appears to be CCR10 dependent (120), CCR8 likely contributes to initial skin tissue homing. Skin resident NK cells specifically express CCR8 (95, 118), and CCR8 expression on specific ILC cells (discussed below in greater detail) is critical for skin homing (127). Though its ligand CCL1 is not expressed by keratinocytes or dermal fibroblasts, low levels of CCL1 on microvascular endothelial cells may initially recruit circulating NK cells (128). CCR8 is also critical for regulating the migration of inflammatory cutaneous dendritic cells from skin to draining lymph nodes in contact hypersensitivity reactions (129). These then trigger skin homing of CCR10^+^ ILCs from circulation to skin-draining lymph nodes (120). Further work is needed to delineate the role of CCL27-CCR10 axis in mediating adhesion and initiating transepidermal migration of cNK cells into skin.

As most of current understanding of NK cell recruitment and migration to and effector function within skin has been garnered from models of cutaneous tissue perturbed by various pathologies, further discussion of this subject will be described through highlighting prior research findings in three general areas of human and murine disease: infection, inflammatory/autoimmune disease, and cutaneous malignancy (**Table 3**) (130–143).

**Table 3:** NK cell homing in cutaneous disease.

	Addressins/pathways
Infection	
Dengue virus	- CLA, CCR5, CXCR6 up-regulation [130 – human]
Leishmaniasis	- CCR10 (KO improves skin disease) [119 – mice]
Vaccinia virus	- Low CLA expression [131 – human]
Cytomegalovirus	- EOMES+ and acquire CD69 [43 – mice]
	- TRAIL-dependent [19 – mice]
Inflammatory/Autoimmune	
Atopic Dermatitis	- CLA+ [125 – human]
	- IL-15 KO increases ILC2 cells & correlates with worse eczema [133 – mice]
	- IL-17 inhibits trNK cell function in mouse eczema herpeticum model & worsens skin disease [132-133 – mice]
Psoriasis	CXCR3, CCR5, - diminished CLA expression [118 – human]
Allergic contact dermatitis	- CCL27-CCR10 axis [123,135 – mice]
	- CXCR3^+^CCR6^+^CCR5^+^ [136 – human]
Hidradenitis suppurativa	- CD38 [137 – human]
Cutaneous malignancy	
nasal type NK cell lymphoma	- CLA+ [138 – human]
Melanoma	- TIM3 expression [139 – human]
	- Decreased ICAM1 & E-selectin [140 – human]
Squamous cell carcinoma	- TGFβ signaling [141-142 – human]
	- Decreased E-selectin [143 – human]

## NK cells in lesional skin

### Infection

As just discussed, leukocytes found in the skin possess a repertoire of homing receptors, including CLA and a number of chemokine receptors (CCR4, CCR6, and CCR10), that facilitate trafficking to sites of inflammation (128). Many ILC2 cells derived from healthy donors also express baseline levels of CLA, CCR10, and CCR4 (43). Chemokine receptor CCR8 facilitates ILC2 migration from the bone marrow to the skin upon trauma to skin tissue (82). During acute infection, CLA expression on circulating vs trNK cells appears to be variably expressed, suggesting a crucial role for CLA in homing to and infiltration of skin in response to specific pathogens. In cutaneous leishmaniasis, for example, CD8^+^ T cells expressed higher levels of CLA within the skin, while circulating NK cells demonstrated reduced CLA expression, compared to those of age-matched controls (131). In contrast, infection with Dengue virus results in increased expression of CLA and other homing receptor (CCR5 and CXCR6) by circulating NK cells (130). It is unclear whether NK-cell mediated response to certain pathogens within the skin plays a role in the resultant tissue damage. For example, accumulation of circulating NK cells with higher cytotoxic capacity (CD56^dim^/CD57^bright^) positively correlated with skin lesion size in patients with cutaneous leishmaniasis (131), although the tissue pathology was thought to be more CD8^+^ T cell driven. Furthermore, CCR10 knock-out mice demonstrated robust and protracted innate immune responses in the skin that phenotypically manifested as increased clearance of cutaneous Leishmaniasis (119).

Are these NK cells recruited to the skin in response to particular infectious agents, already present based on prior cutaneous microbe sensitization, or some combination of the two? Although relatively unexplored in skin, recent works have revealed that tissue resident ILC cells can be mobilized in response to specific inflammatory cues and migrate to other organs (94, 144–146). A study in which CD45.1^+^ and CD45.2^+^ parabiotic mice were surgically connected for 30–40 days to achieve complete blood chimerism demonstrated that >95% of ILC cells (regardless of subset) within these tissues were of host origin (147). Notably, the one exception was ILC1 cells (Eomes^-^Nk1.1^+^) in lung and peripheral blood, which originated from both parabionts. Again, though, none of these experiments evaluated the skin.

A few studies have, however, looked at NK cell homing in response to local cutaneous infection. In murine models, following intradermal infection with vaccinia virus or *Staphylococcus aureus*, NK cells were immediately recruited to the site of skin infection and retained for up to 80 days post-infection (14). While cNK cells are phenotypically Eomes^+^/CD49a^-^, a number of recent studies in various tissues (uterus, salivary glands, etc) have demonstrated establishment of a Eomes^+^CD49a^+^ tissue-resident NK cell population (65, 148, 149). These are phenotypically and ontogenically distinct from resident ILC1 Eomes^-^CD49a^+^ cells within these tissues (149, 150). Similarly, Torcellan et al. demonstrated in skin that cNK cells are recruited to sites of cutaneous infection and subsequently undergo transcriptional reprogramming that render their expression profile similar to that of trNK cells (Eomes^+^CD49a^+^), with some evidence of memory-like features (CD69^+^) (14). As expected, when challenged near the previous site of infection with the same pathogen, a more robust effector response was mounted than that observed during the initial infection (14). Similarly, a population of trNK cells was recruited and expanded within murine salivary glands following CMV infection (151). Unexpectedly, these trNK cells were shown to eliminate anti-viral CD4^+^ cells in a TRAIL-dependent fashion (38), ultimately preventing the development of Sjogren’s syndrome, as compared with the control group. This observation suggests a more immunoregulatory role for mucocutaneous trNK cells in this context. Notably, Schuster et al. demonstrated that the trNK cell population that developed in these salivary glands in response to CMV infection was distinct from the populations present in infection-naïve mice (151). Given the complex relationship of human skin to its innumerable commensal and pathogenic microbes, it is unsurprising that immune cells responsible for bridging innate and adaptive immunity would be highly responsive and adaptable on a pathogen-specific basis. Construction of this highly variable adaptive programming likely begins during pre- and early post-natal development (87).

Finally, the critical role of NK cells in combating infections with cutaneous manifestations is abundantly evident in patients with low NK cell levels and/or NK cell deficiencies. NK cells especially play a role in eradication of the herpesvirus family, including herpes simplex virus (HSV), varicella zoster virus (VZV), Cytomegalovirus (CMV), Epstein-Barr virus (EBV), and human papilloma virus (HPV) (152–159). Patients with DOCK8 immunodeficiency syndrome are particularly susceptible to HPV infections, a common cause of cutaneous warts and increased HPV-driven SCC (160). EBV infections with mucosal and skin findings are increased in patients with Chediak Higashi syndrome (161). In addition to skin, teeth and hair abnormalities, patients with NEMO (NFκB) deficiency syndrome, who have both low NK cell and antibody function, are unable to mount a sufficient response to numerous bacterial and viral infections (162). Herpesvirus infections are also common in other immunodeficiency syndromes with low NK cell levels, including Wiskott-Aldrich (163), familial hemophagocytic lymphohistiocytosis (164–166), and Hermansky-Pudlack syndrome (167, 168). NK cell deficiency in humans is also associated with an increase in disseminated HSV and VZV infections (153, 169, 170). VZV infection in particular triggers CCR4 and CLA activation in infected NK cells, initiating their migration directly to the skin (154). Viruses like human immunodeficiency virus (HIV) and Kaposi’s sarcoma-associated herpesvirus down-regulate MHC class I molecules on the surface of infected cells to evade cytotoxic T cells, making them more susceptible to NK cell-mediated responses (171), but unfortunately NK cell activity in HIV infection is often defective (172, 173). The innate anti-viral function of NK cells presents a promising therapeutic avenue for exploitation of direct cellular therapy in cutaneous infectious disease.

## Inflammatory/autoimmune

**Figure 1:**
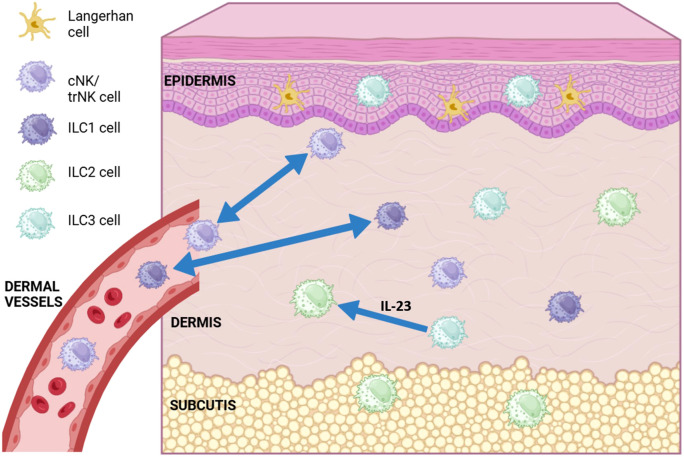
Diagram of conventional (cNK), tissue resident (trNK) NK cell and ILC (ILC1, ILC2, ILC3) subsets found in different layers of the skin. ILC1 and cNK cells have been shown to migrate back and forth between the dermis and dermal vasculature (blue double-headed arrows). ILC3 cells have been show to transdifferentiate into ILC2-like cells in the presence of IL-23 (blue arrow). Langerhans cells are the most abundant immune cell type found in the skin, most commonly in the epidermis. (Figure created using Biorender.com).

Skin ILCs are felt to significantly contribute to the maintenance of tissue homeostasis, and their imbalance is implicated in a number of autoimmune and inflammatory diseases, such as atopic dermatitis (AD) and psoriasis (174). As previously mentioned, each of the ILC subgroups within the skin (as in other tissues) appear to mirror their T helper cell counterparts (175). For example, ILC1 cells are considered the innate counterparts of CD4^+^ Th1 cells, ILC2 cells have a role in atopic dermatitis (synonymous with eczema) akin to the Th2 subset, and ILC3 cells, which produce IL-17, may be relevant for psoriasis (91, 92, 176, 177). ILC2 is normally expressed in the dermis and subcutis, whereas ILC3/LTi-related genes are found primarily in the epidermis (178–180). ILC2 and ILC3 cells are mostly considered tissue resident, though as previously discussed, can be induced to migrate under inflammatory conditions. ILC1 cells traffic back and forth between circulation and the lymph nodes, dependent on CD62L and CCR7 (94) (**Figure 1**).

Interestingly, the basal level and subtype of ILCs within skin may be directly influenced by environmental exposures, as discussed above with cutaneous infection-triggered immune responses. For example, ILC2 cells produce type 2 cytokines (IL-4, IL-5, IL-9, IL-13) (181–184) and are overexpressed in both mouse and human atopic lesional skin (43). Moreover, evidence exists of *additional* ILC2 recruitment to these inflammatory sites, mediated by skin-specific expression of IL-33 (185). Notch signaling may play a significant role in this ILC2 plasticity in its conversion from a resting to pro-inflammatory state (186). Blocking Notch signaling via γ-secretase (a pan-Notch inhibitor) in a mouse model treated with recombinant IL-25 to induce inflammatory ILC2 populations abrogated their cytokine responsiveness and ability to transdifferentiate into an ILC3-like state (186). Addition of Notch ligands to isolated resting ILC2 cells *in vitro* rescued this phenotype of maintaining ILC2 expression of *GATA3* while acquiring an ILC3-like ability to secrete IL-17 in its pro-inflammatory state. Atopic lesional skin specifically demonstrates an influx of granzyme positive NK cells in the dermis, with homing receptor CLA^+^ expression being especially prevalent in patients with severe disease (125). It is unclear, however, whether cNK cells recruited to the skin contribute to inflammatory pathogenesis, or instead help *limit* type 2 inflammatory responses mediated by local/previously established tissue resident ILC populations. IL-15 knock-out mice, which are NK cell deficient and clinically manifest a chronic eczematous dermatitis phenotype, demonstrate an increase in ILC2 cells and eosinophils within both lesional skin and skin-draining lymph nodes (133). Further, peripheral blood and lesional skin of patients with AD have a global reduction in NK cells, but also demonstrate decreased total CD56^dim^ mature effector NK cells, which recovered following patient treatment with Dupilumab (IL-4 and IL-13 inhibitor) (133). This suggests a distinct role for cNK cell recruitment and skin infiltration in attempting to limit Th2 inflammatory response within disease-specific contexts.

Eczema herpeticum (EH) is one of the gravest and most feared complications in patients with atopic dermatitis. Eczematous mice intradermally injected with HSV1 virus rapidly develop EH; lesional skin from these mice was found to have reduced NK cell activity compared to non-eczematous mice injected with the virus (134). Adoptive transfer of healthy NK cells into these eczematous mice the day prior to viral infection, however, decreased their EH scores. Interestingly, skin lesions of eczematous mice infected with the vaccinia virus have increased numbers of NK cells compared to non-eczematous mice infected with the same virus; however, NK cell cytotoxic activity (measured by expression of granzyme B, perforin, and IFNγ) is significantly reduced in the eczematous mice (134). Adoptive transfer of healthy NK cells via intravenous injection into eczematous mice prevented development of skin lesions following vaccinia infection, and antibody-mediated IL-17A neutralization in eczematous mice delayed skin lesion onset after virus infection (134). Furthermore, this reduction in development of skin lesions was abrogated by αAGM1 antibody-mediated depletion of NK cells prior to IL-17A neutralization, suggesting IL-17A directly affects NK cell activity in virally-infected eczematous mice.

Parabiotic mouse experiments have shown that ILCs in the gut, lung, and skin are maintained and expanded locally in physiologic and pathogenic conditions with only minor contributions from circulating progenitors (147). What then, is the degree of plasticity between the various trNK and ILC subsets? Although ILC2 cells are the predominant ILC subtype found within healthy dermis, ILC3 cell populations are predominant in this layer in human and mouse psoriatic skin (92). Psoriatic plaques on human skin have also demonstrated an infiltrating immune cell population comprised of CD3^-^CD56^+^CD16^-^CD158b^-^ NK cells, all of which are localized to the mid and papillary dermis (118). These skin-infiltrating NK cells express high levels of CXCR3 and CCR5 chemokine receptors, release abundant IFNγ following stimulation, and fail to express the skin-homing CLA antigen (118). Absence of the chemokine CCL27 in a knock-out mouse model results in an overactive cutaneous inflammatory response in the well-established model of imiquimod-induced psoriasis (123).

While much of the current literature regarding NK cells within inflammatory cutaneous conditions focuses on psoriasis and atopic dermatitis, where their numbers are greatly reduced and/or skewed to one specific ILC phenotype, there have also been documented in studies of immune subpopulations in several other diseases, where NK cells seem to play a more inflammatory role. Lichen planus (LP), whose damage is hallmarked by lymphocyte-induced vacuolization of basal keratinocytes at the junction between the epidermis and dermis, demonstrates a high number of CD3^-^CD56^high^CD16^-^ NK cells in early LP lesions (136). Skin NK cells isolated from these lesions were negative for inhibitory receptors (KIR receptors CD158a and b), but highly positive for perforin and the activating receptors NKG2D and NKp44; additionally, these cells possessed the ability to secrete IFNγ (136).

Another common inflammatory cutaneous disease is allergic contact dermatitis (ACD). While Rag -/- mice (lacking lymphocytes) are unable to mount a contact hypersensitivity response, adoptive transfer of NK cells from sensitized donors restores this response, independent of B or T cells (135). NK cells accumulate abundantly in lesional skin of patients with ACD and secrete IFNγ in the presence of hapten-driven T cells *in vitro* (136). Interestingly, ILC1 cells sensitized in the skin draining lymph nodes in a mouse ACD model were then recruited to the liver, where they were retained and could be reactivated upon hapten re-exposure (20). Direct skin hapten challenge after this pre-requisite sensitization then mediated early recruitment (within the first 24 hours) of NK cells to the skin and skin draining lymph nodes (187). ILC1, ILC2, and ILC3 cell numbers then increased after this initial response, with subsequent decreases in cNK cells within the skin. Localization of this hapten-memory specific NK cell subpopulation to the liver suggests that some NK cells possess memory-like properties generally thought to be more associated with adaptive immune cell populations (135). In contrast to T-cell mediated hypersensitivity responses, however, NK cell induced inflammation is more transient and less inflammatory than that mediated by CD8^+^ T-cells (188).

Specific autoimmune inflammatory skin conditions associated with reduced numbers of NK cells include hidradenitis suppurativa (HS), lupus, and systemic sclerosis (189–191). Expression of CD38, a glycoprotein found on the surface of NK, B, and T cells that is an important mediator of inflammation, is often dysregulated in HS (137). ILC1 and 3 subsets are increased and ILC2 decreased in peripheral blood of patients with systemic lupus erythematosus (SLE) and in patients with HS compared to normal controls (192–194). While these studies do not directly examine the role of skin-infiltrating NK cells within these cutaneous disease states, cNK cell dysregulation within SLE is well-established. Not only are NK cell populations decreased in peripheral blood of patients with SLE compared to controls (195), but co-culture of serum of patients with SLE with NK cells from healthy controls actually inhibits NK cell activity (196). Interestingly, the degree of NK cell functional inhibition directly correlates with SLE disease activity (196). Better characterization of cNK cell recruitment and contribution to inflammatory and autoimmune skin diseases may further our understanding of disease-specific pathogenesis.

## Cutaneous malignancy

NK cells are programmed to immediately and aggressively respond to viruses and tumor cells, so better characterization of the role of NK cells within cutaneous malignancy provides a promising potential area for therapeutic intervention. In general, impaired NK cell function correlates with high tumor stage and worse prognosis in a number of solid tumors [reviewed by 57]. For example, NK cell tumor infiltration is an independent predictor of progression free survival in GI stromal tumors and pulmonary adenocarcinoma (197, 198). Most of the work investigating the role of NK cells in cutaneous malignancy to date has understandably focused on melanoma, the most deadly skin cancer. Patients with melanoma have an enriched population of CD56^bright^CD16^dim^ NK cells (199), which correlates with reduced overall and progression free survival (200). Some studies, however, have shown enrichment of CD56^dim^KIR^+^CD57^+^ highly cytotoxic NK cells in tumor-infiltrated lymph nodes in melanoma patients (201, 202), suggesting that arrest of NK cell cytotoxic efficacy likely occurs after skin tumor infiltration. It appears that trNK cells within the skin inherently lack direct tumor killing properties without cytokine-mediated licensing. Further support for this hypothesis comes from the finding that peripheral blood-isolated NK cells display an innate ability to kill both murine and human melanoma cells *in vitro* (203, 204), yet NK cells isolated directly from healthy human skin require culture in the presence of IL-2 to induce cytolytic activity against melanoma cells (95). It was also recently demonstrated that IL-2-expanded NK cells suppress cSCC cell survival and tumor growth both in 3D cell culture models as well as xenograft cSCC tumors in NSG mice (205). Treatment with these expanded NK cells resulted in a dose-dependent reduction in spheroid growth and Matrigel invasion assays, with an associated increase in apoptosis signaling pathways (205). Additionally, expression of leukocyte homing receptors and subsequent adhesion receptors (especially E-selectin and ICAM1) is reduced and/or dysregulated in malignant melanoma (140) and cutaneous squamous cell carcinoma (cSCC) (143). Interestingly, RNAseq data gene set scoring of tumors from patients with metastatic melanoma demonstrated that improved survival/prognosis was associated with increased NK cell tumor infiltration (206).

In contrast, cutaneous NK cell lymphomas appear to pose the opposite threat: too many activated skin-infiltrating NK cells. A number of case series and smaller studies on skin tissue from patients with this rare cutaneous lymphoma have shed an intriguing light on NK cell homing to and effector function within the skin. In particular, two cases of aggressive non-nasal extranodal NK cell lymphomas associated with EBV infection demonstrated early dissemination to the skin, liver, spleen, and BM (207). These tumors were CD56^+^CD16^+^, and expressed CLA, granzyme B, and TIA-1. In a study of 52 cases of NK-cell lymphoma, CLA expression was highly expressed in cases with a *cutaneous* component, compared to their non-cutaneous counterparts (138). The CLA^+^ group also had a significantly worse prognosis compared to the CLA^-^ group, regardless of the tumor’s primary site or clinical staging (138). High CLA expression levels are also seen in NK-like T-cell lymphoma (208). Given its role in recruitment of circulating lymphocytes during active inflammation (including NK and T cells), these findings likely reflect a CLA mediated increase in NK cell infiltration and retention within cutaneous tumor tissue. Better characterization of this homing mechanism may provide contextual insights into when and why NK cells are recruited to and retained within inflammatory vs malignant skin tissue, and how their phenotype and function are affected upon arrival.

While great success has been achieved with the use of NK cell adjunct therapy in hematologic malignancies (209–219), it is well established that NK cells are rapidly rendered dysfunctional upon entry to the tumor microenvironment (TME) in several solid tumor malignancies (220). Melanoma-associated fibroblasts reduce NK cell cytotoxic capacity and function (221, 222), so it is unsurprising that a small clinical trial testing adoptive transfer of NK cells infused into patients with progressive stage IV melanoma saw no clinical response (223). A number of studies have demonstrated a role for immunotherapy in restoring NK cell function within the TME, with the degree of responsiveness to immunotherapy directly associated with baseline cNK cell presence before and during treatment. Survival in a cohort of patients with malignant melanoma treated with anti-CTLA-4 correlated with both low expression of the inhibitory receptor TIM-3 on circulating T and NK cells during and prior to treatment, as well as increased frequency of mature NK cells (CD3^-^CD56^dim^CD16^+^) during therapy (224). Treatment of patients with melanoma with ipilimumab induced a significant increase in CD56^dim^, but not CD56^bright^ NK cells in the periphery (225). Importantly, there appears to be a role for circulating NK cells in limiting pulmonary seeding of melanoma metastasis and in recruitment of T cells directly to metastatic foci (226). Ongoing efforts include a Phase I/II clinical trial infusing *ex-vivo* expanded allogeneic universal donor NK cells in combination with Temozolomide as a lymphodepleting agent in patients with melanoma and brain metastasis (NCT 05588453).

Most NK cell research in cSCC has been performed on head and neck SCC (HNSCC), which is commonly HPV-driven (227). Cetuximab inhibits EGFR pathway signaling and activates innate immunity, partially via stimulation of NK cells in HNSCC (228). Co-treatment using PD1 blockade with stimulated NK cells (via monoclonal antibody-mediated blocking on the NK inhibitor receptor NKG2A) in a phase II trial treating head and neck SCC has shown encouraging results (229). Additionally, the PD-1 blocking antibody cemiplimab, which enhances NK cell intra-tumoral activity, has demonstrated promising treatment responses in metastatic cSCC (230–232).

Significantly, all of the studies discussed above regarding melanoma and cSCC have focused on NK cell levels circulating in the blood or in tumor-infiltrated lymph nodes. While little has been done to investigate the role of trNK cells directly within the TME of these cutaneous malignancies, a few studies demonstrate the positive effect of functional NK cells within skin tumors, as previously discussed (206, 233). Mechanistically, NK cell production of *Flt3lg* within the TME of melanoma tumors directly affects the level of intra-tumoral stimulatory dendritic cells (antigen presenting cells that are important for infection and tumor defense within skin). This NK cell infiltration not only correlated with increased overall survival in patients with melanoma, but also predicted positive responsiveness to anti-PDL1 immunotherapy (233). As mentioned previously, NK cells typically circulate in peripheral blood, but can be recruited via stress signals from pathogenic cells, such as directly to tissue (i.e., skin) (68). Though infiltrated with an abundance of NK cells, human cSCC-associated NK cells have impaired cytolytic function and IFN-γ production, which can partially be restored upon *in vitro* stimulation (234).

So, why are cNK cells homing to the skin for anti-tumoral response losing their cytolytic capacity? In addition to the prior discussion regarding a potential contribution of CD16 shedding in this process, a number of studies demonstrate a critical role for TGFβ signaling in direct suppression of NK cell activity and anti-tumor function within the TME. TGFβ1, a cytokine critical for wound healing in the skin (235), inhibits NK cell-mediated killing of dendritic cells through selective inhibition of NKp30 expression on NK cells (236). There is also evidence for a role of TGFβ signaling in mediating conversion from cNK cells (CD49a^-^CD49b^+^Eomes^+^) into a tissue-resident ILC1-like cell population in salivary glands and intestinal mucosa (65, 237). Suppression of this TGFβ signaling in NK cells enhances their ability to limit metastases in multiple tumor models in mice (including melanoma) through disinhibition of the mTOR pathway (238). In contrast, absence of SMAD4 in ILC1 and NK cells in mice caused NK cells to acquire an ILC1-like gene signature; these cells were unable to control tumor metastasis or viral infection (237). These findings suggest a role for canonical SMAD4 in restraining non-canonical TGFβ signaling via cytokine receptor TGFβR1 in NK cells (237).

We previously described a method for propagating large numbers of clinical-grade NK cells *in vivo* with IL-2 and irradiated K562 feeder cells expressing membrane-bound IL-21 and 4-1BBL (239, 240). More recently, we reported a modification of that method that enhances NK cell function and overcomes TGFβ-induced suppression (referred to as TGFβ “imprinting” by serially stimulating the NK cells with TGFβ during the expansion process (241). The addition of TGFβ during NK cell propagation impairs neither fold expansion nor viability of the final expanded NK cell product, but the resulting TGFβ imprinted (TGFβi) NK cells exhibit high cytotoxicity and a pro-inflammatory hypersecretion of IFN-γ and TNFα in response to tumor targets (241). Moreover, these cells significantly downregulate SMAD3 at the transcriptional level, resulting in resistance to suppression by TGFβ. Importantly, this cytokine hypersecretion persists for one month after removal of TGFβ (241), suggesting that TGFβi NK cells may retain their enhanced cytokine secretion *in vivo*, where IFNγ and TNFα secretion can then stimulate adaptive immunity and sensitize tumors to NK cell killing (27). Given the previously discussed evidence for TGFβ imprinting in mice to an ILC1-like phenotype (65, 237), we postulate that the unique characteristics of this modified NK cell product would enable these *ex-vivo* imprinted cells to persist following direct injection into skin tumors with an ILC1-like or skin tissue resident phenotype. Their acquired resistance to TGFβ and enhanced cytotoxic properties would then enable these cells to overcome the inherently suppressive TME of the cutaneous malignancy, and also mediate robust anti-tumoral activity. To this end, further pre-clinical and exploratory clinical studies are currently underway.

## Discussion

### Missing pieces/next steps/harnessing NK cells

One driving challenge to cohesion within the NK cell literature is the complex heterogeneity in defining NK cells dependent on the specific tissue and disease of interest. It appears universally accepted that NK cells are broadly defined as CD56^+^CD3^-^ (differentiating them from T lymphocytic cell populations). However, until recently, there has been no standardized panel for describing NK cells. Some groups have previously described them in terms of being CD56^bright^CD16^-^ vs CD56^dim^CD16^+^, having lytic or IFNγ secretory functions, circulating vs being tissue resident, or even based on their secretion of and response to specific cytokines (242, 243). For example, NK-22 cells were identified as a subpopulation found in mucosa-associated lymphoid tissues (i.e., Peyer’s patches and tonsils) that secrete IL-22 in response to IL-23 (32). Activated NK-22 cells *in vitro* stimulated epithelial cells to secrete IL-10 and to proliferate (32). Interestingly, a skin homing population of memory T-cells (CLA^+^CCR10^+^CCR6^+^CCR4^+^), referred to as “Th22,” comprise the majority of T lymphocytes isolated from psoriatic skin lesions (244). Given their known pathogenic role of secreting IL-22 in response to IL-23, NK-22 cells are likely also implicated in psoriasis (245, 246), though this has yet to be confirmed. Do these NK-22 cells truly represent a distinct subpopulation of NK cells? Or is this evidence that NK cells are marked by a degree of plasticity that enables immense adaptability in a pathogen-driven and microenvironment-specific context?

As previously discussed, NK cells circulating in peripheral blood were most recently characterized into three distinct subsets: NK1, NK2, and NK3 cells, respectively, that are each characterized by a unique portfolio of protein and transcriptional programing (53). The range of plasticity of ILC subsets and trNK cell populations within skin is still unclear: do NK cells entering tissues “transform” into ILC1-like cells, or do these ILC and trNK subsets represent distinct immune cell entities? Park et al. demonstrated NK-to-ILC1-like transdifferentiation in response to Toxoplasma gondii infection, and this population expanded within both affected tissues as well peripheral circulation (247). Conversely, in the skin, there is evidence for adaptation of circulating Eomes+ NK cells into CD69+Eomes+ resident NK cells, without loss of Eomes or conversion into ILC1s (14). These findings suggest that tissue-resident NK cells in the skin are a distinct population arising from circulating cNK cells, which at times may take on a ILC1-like phenotype, but are ontogenically distinct from ILC1 cells.

Additional questions to be answered within the skin tissue NK cell literature include understanding the two-faced role for NK cells in mediating host immune response, while also preventing host T cells from excessive activation and causing autoimmune damage to the specific tissues it should be protecting from pathogens. Many of the studies investigating cutaneous inflammatory and autoimmune processes have focused on T cell populations/subsets, with more recent data identifying ILC subsets within the skin that “mirror” their T helper cell counterparts, including in response to specific transcription factors (i.e., Th2 and ILC2 predominance in lesional skin of human and murine atopic dermatitis) (79, 92, 122, 244, 248). Given the critical role for NK cells as a bridge between innate and adaptive immunity, there is a need to further elucidate the role for disequilibrium in NK cell recruitment and function in the skin as it pertains to the pathogenesis of cutaneous autoimmune and inflammatory processes.

From a clinical or translational standpoint, what further implications do these findings carry? NK cells play a negligible role in graft rejection in skin and other organs and tend not to mediate graft-vs.-host disease (GvHD), making them an ideal effector tool for cellular therapy (249, 250). Additionally, NK cells are not thought to be involved in GvHD in stem cell transplants, with the exception of a single paper reporting significant GvHD following NK cell infusions (251). Understanding how trNK cells are recruited, activated, and regulated within different contexts and how they communicate with microorganisms and other tissue-resident immune cells, including surrounding stroma (epithelial cells, fibroblasts, neurons), may provide insights that would enable development of unique therapeutic strategies. For example, a recently published study describes a unique strategy to co-op innate immunity as a topical therapeutic approach in psoriasis to modulate pathologic inflammatory skin ILCs (252). Additionally, Mack et al. observed NK cell deficiency with an associated increase in type 2 cutaneous inflammation in patients with atopic dermatitis [Mack 2020]. The AD-like phenotype in their mouse model was significantly improved via treatment with an IL-15 superagonist, which promotes NK cell proliferation and survival. Direct NK cellular therapy also presents exciting opportunities for intervention within cutaneous malignancy, including accompaniment of a markedly improved side effect profile compared with that currently experienced with immune checkpoint inhibitor immunotherapies (253). Overall, these and other emerging strategies to enhance NK cell function within various skin pathologies offer promising therapeutic potential for a wide range of cutaneous diseases.

## Conclusion

As early as the pre-natal period, human skin serves as barrier tissue that is confronted with and must adapt to a unique and highly individualized array of pathogens, sensitizing agents, and environmental exposures. Though the largest and most accessible organ providing this critical defense, skin is frequently overlooked in the NK cell literature, likely in part due to the heterogeneity in defining trNK cell origin and subsets. We submit that skin represents one of the secondary lymphoid organs of the human body, and that further characterization of skin trNK cells will reveal a highly plastic and adaptable immune cell population that is critical not only for skin immune development and maintenance of homeostasis, but also with the potential to provide unique therapeutic avenues for treatment of cutaneous infection and inflammatory and malignant diseases.
